# Interobserver Reliability of Magnetic Resonance Imaging of Sacroiliac Joints in Axial Spondyloarthritis

**DOI:** 10.3390/life12040470

**Published:** 2022-03-23

**Authors:** Anca Emanuela Mușetescu, Anca Bobircă, Florin Liviu Gherghina, Alesandra Florescu, Florin Bobircă, Paulina Lucia Ciurea, Cristina Criveanu, Alice Muscă, Lucian Mihai Florescu, Ioana Andreea Gheonea

**Affiliations:** 1 Department of Rheumatology, University of Medicine and Pharmacy of Craiova, 200349 Craiova, Romania; anca.musetescu@umfcv.ro (A.E.M.); paulina.ciurea@umfcv.ro (P.L.C.); cristina.criveanu@umfcv.ro (C.C.); 2 Department of Rheumatology and Internal Medicine, Carol Davila University of Medicine and Pharmacy, 050474 Bucharest, Romania; anca.bobirca@umfcd.ro; 3 Department of Physical Medicine and Rehabilitation, University of Medicine and Pharmacy of Craiova, 200349 Craiova, Romania; florin.gherghina@umfcv.ro; 4 Department of General Surgery, Carol Davila University of Medicine and Pharmacy, 050474 Bucharest, Romania; florin.bobirca@umfcd.ro; 5 Department of Internal Medicine and Rheumatology, Dr. I. Cantacuzino Clinical Hospital, 011437 Bucharest, Romania; andreea-alice.musca@spec.umfcd.ro; 6 Department of Radiology and Medical Imaging, University of Medicine and Pharmacy of Craiova, 200349 Craiova, Romania; ioana.gheonea@umfcv.ro

**Keywords:** axial spondyloarthritis, magnetic resonance imaging, sacroiliitis, interobserver reliability

## Abstract

Introduction: Axial spondyloarthritis (axSpA) is characterized by damage to the axial skeleton and entheses, and is often associated with extra-articular manifestations, in the presence of the human leukocyte antigen (HLA) B27. The aim of our study is to assess the performance of rheumatologists in interpreting the inflammatory and structural damage to sacroiliac joints, in comparison to radiologists. Material and Methods: The present study included a total of 34 patients diagnosed with axSpA, according to the Assessment of SpondyloArthritis International Society (ASAS) criteria for axSpA, examined from January 2021 to November 2021 in the Departments of Rheumatology and Radiology and Medical Imaging of the University of Medicine and Pharmacy of Craiova. All patients underwent physical examination, laboratory tests, and magnetic resonance imaging (MRI) of the sacroiliac joints. The images were interpreted by a senior radiologist (SR), a junior radiologist (JR), a senior rheumatologist (SRh), and a junior rheumatologist (JRh), who were blinded to the clinical and paraclinical data. Results: The overall κ was 0.7 for the JR (substantial agreement), 0.707 for the SRh (substantial agreement), and 0.601 for the JRh (moderate agreement), in comparison with the SR. Regarding the overall inflammatory changes, the SRh and JR were proven to have substantial agreement (κ = 0.708 and 0.742, respectively) with the SR, while the JRh was proven to have moderate agreement (κ = 0.607). The structural damage observed by the JR showed substantial agreement (κ = 0.676) with the SR, while the SRh and JRh had substantial and moderate agreement (κ = 0.705 and 0.596, respectively) with the SR. Conclusions: Our study showed substantial agreement between the senior radiologist, senior rheumatologist, and junior radiologist, and moderate agreement with the junior rheumatologist.

## 1. Introduction

Axial spondyloarthritis (axSpA) is characterized by damage to the axial skeleton (spine and sacroiliac joints) and entheses, and is often associated with extra-articular manifestations, such as ocular, cardiac, pulmonary, or renal involvement, in the presence of the human leukocyte antigen (HLA) B27 [1,2,3].

The term axial spondyloarthritis includes both the radiographic and non-radiographic forms, in which case sacroiliitis can be objectified by magnetic resonance imaging (MRI) only, and not by conventional radiographs. This form of the disease is called non-radiographic axial spondyloarthritis (nr-axSpA), and may or may not progress to ankylosing spondylitis (AS) [4,5].

The hallmark of sacroiliitis, from a clinical point of view, is represented by sacroiliac joint pain. Furthermore, while sacroiliac discomfort is often restricted to the buttocks and lower lumbar areas, some patients experience pain radiating to the groin, lower abdomen, trochanter, and even lower leg. Joint palpation and provocation tests, aimed at inducing pain while straining the sacroiliac joints, are part of the physical evaluation of the sacroiliac joint [6,7].

However, at least in the early stages of sacroiliitis, alterations in the sacroiliac joints, detected on radiographs, are not sensitive or specific enough. In many individuals with axSpA, it may take years of clinically obvious illness before clear abnormalities of the sacroiliac joints become detectable on standard radiographs [8,9].

MRI can identify joint inflammation in its early stages, before structural damage develops, thanks to its good contrast resolution. With potentially successful therapy, applicable to a limited window of opportunity for disease control, MRI has become a dominating diagnostic technology, and, at the same time, was incorporated in the axSpA classification criteria, thus becoming the cornerstone of SpA diagnosis [10,11].

Sacroiliitis is usually bilateral and symmetrical. Initially, it involves the lower two-thirds of the sacroiliac joint space. The progression of the erosive process results in pseudo-enlargement of the sacroiliac joint space with bone sclerosis, followed by complete fusion of the sacroiliac joints.

In daily practice, MRI images are interpreted by radiologists, and the readings are presented to the primary care physician or to the rheumatologist. However, a rheumatologist with special training should be able to interpret musculoskeletal MRI examinations. The interobserver agreement, defined as the difference in the measurements between radiologists and rheumatologists, should be further tested. It is not clear whether the assessment criteria for sacroiliitis are reproducible among rheumatologists and radiologists with different levels of experience [12,13].

The aim of our study is to assess the performance of rheumatologists in interpreting the inflammatory and structural damage to sacroiliac joints, in comparison to radiologists.

## 2. Materials and Methods

### 2.1. Patients

The present study included a total of 34 patients diagnosed with axSpA according to the Assessment of SpondyloArthritis International Society (ASAS) criteria for axSpA, examined from January 2021 to November 2021 in the Departments of Rheumatology and Radiology and Medical Imaging of the University of Medicine and Pharmacy of Craiova. The inclusion criteria were as follows: age >16 years, diagnosis of AS, and symptoms of sacroiliac joint pain lasting for more than 6 months. The exclusion criteria included the presence of any other rheumatic disease, infection, or trauma to the pelvic region. All patients expressed their agreement to be a part of this study. The study was approved by the local ethics committee of the University of Medicine and Pharmacy of Craiova (Registration no. 210/08.12.2021), according to the European Union Guidelines (Declaration of Helsinki).

### 2.2. Demographic Characteristics and Assessment of Clinical, Laboratory, and Imaging Data

All patients underwent physical examination, laboratory tests, and MRI of the sacroiliac joints.

The clinical assessment included specific maneuvers for the sacroiliac joints, such as Patrick’s maneuver. Patrick’s maneuver, or the FABER test, was performed by having the leg flexed, while the thigh was abducted and externally rotated. If pain was perceived anteriorly on the ipsilateral side, it suggested hip joint dysfunction on that side. If pain was evoked posteriorly, on the contralateral side around the sacroiliac joint, it was likely caused by sacroiliac joint dysfunction. The patient’s pain and severity were assessed with a visual analog scale (VAS) of 100 mm. Standardized scores, such as the Ankylosing Spondylitis Disease Activity Score (ASDAS), Bath Ankylosing Spondylitis Disease Activity Index (BASDAI), and Bath Ankylosing Spondylitis Functionality Score (BASFI), were used in order to assess disease activity in the group of patients.

The evaluated biological parameters consisted of complete blood count (CBC), liver enzymes, serum creatinine, erythrocyte sedimentation rate (ESR), with a normal range <10 mm/h, and C-reactive protein (CRP), with a normal range <5 mg/L.

All MRI examinations of the sacroiliac joints were performed on a Philips Ingenia 3 Tesla device available in the Department of Radiology and Medical Imaging of the University of Medicine and Pharmacy of Craiova. The MRI protocol for evaluating the sacroiliac joints consisted of coronal oblique T1, T2 (without fat suppression), short tau inversion recovery (STIR) sequences oriented parallel to the sacrum, and axial oblique T2 spectral attenuated inversion recovery (SPAIR) positioned perpendicularly to the sacrum. We evaluated the presence of inflammatory changes (bone marrow edema, synovitis, and enthesitis) and structural damage (subchondral sclerosis, erosions, backfill, fat metaplasia, and joint space narrowing/ankylosis).

The images were interpreted by a senior radiologist (SR), a junior radiologist (JR), a senior rheumatologist (SRh), and a junior rheumatologist (JRh), scored independently without knowledge of the other interpretations. In addition, the scans were anonymized, with no clinical and paraclinical patient data. The SR and SRh have 15 years of experience in radiology and rheumatology, respectively. They both have a particular interest in musculoskeletal imaging, specifically in rheumatic diseases, having attended multiple European Alliance of Associations for Rheumatology (EULAR) and ASAS courses on SpA imaging. The JR and JRh are trainees of the seniors, with 5 years of experience in their chosen specialties.

### 2.3. Statistical Analysis

Statistical analyses of the data were performed using SPSS Software version 20 for Windows. The relationship between the variables was analyzed using the unpaired t-test and the Pearson/Spearman’s coefficient for evaluating correlations. Values less than 0.05 for p were considered to be statistically significant. The summary statistics of the mean ± standard deviation (SD) are presented for continuous variables. The agreement between observers was calculated using cross-tabulation expressed in Cohen’s kappa (κ). The value of kappa was interpreted according to Landis and Koch as follows: <0—poor agreement; 0.0–0.20—slight agreement; 0.21–0.40—fair agreement; 0.41–0.60—moderate agreement; 0.61–0.80—substantial agreement; 0.81–1.00—almost perfect agreement [14].

## 3. Results

The study included 34 patients diagnosed with axSpA (29 males and 5 females), with a mean age of 39.14 ± 10.31 years.

The patients had either only axial involvement, as in 32.25% of the cases, or both axial and peripheral joint involvement, as in 67.75% of the cases. The genetic mark of axSpA, HLA-B27, was positive in 76.47% of the patients.

The disease activity, according to ASDAS-CRP, was low in 8.83% of the cases, and high and very high in 2.94% and 88.23% of the cases, respectively. Regarding BASDAI, the inactive and active disease was described in 5.88% and 94.11% of the patients, respectively.

The descriptive parameters of the patients enrolled in the study are presented in Table 1.

**Figure 1:**
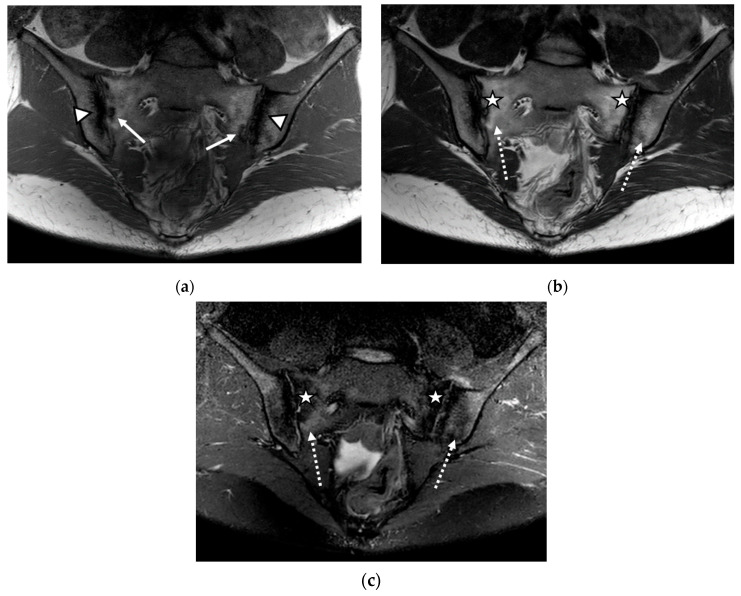
The magnetic resonance imaging (MRI) examination of the sacroiliac joints revealed narrowing of the sacroiliac joint spaces with bilateral marginal bone erosions (continuous arrow), bilateral subchondral sclerosis (arrowhead), and increased fat metaplasia (star), with minimal diffuse adjacent bone marrow edema (dotted arrow): (**a**) T1-weighted sequence; (**b**) T2-weighted sequence; (**c**) short tau inversion recovery (STIR) sequence.

**Figure 2:**
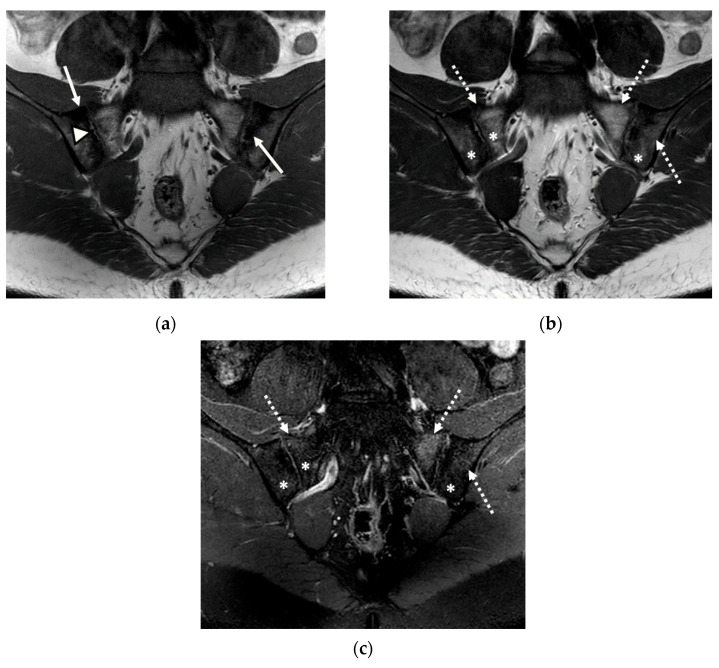
The MRI scan of the sacroiliac joints indicated bilateral narrowing of the sacroiliac joint space, associated with bilateral subchondral sclerosis of the iliac bones (continuous arrow); bilateral fat metaplasia (asterisk), mostly affecting the iliac bones and the right part of the sacrum; backfill of the right sacroiliac joint space (arrowhead); diffuse bone marrow edema (discontinuous arrow), affecting the sacrum and the left iliac bone: (**a**) T1-weighted sequence; (**b**) T2-weighted sequence (without fat suppression); (**c**) STIR sequence.

**Figure 3:**
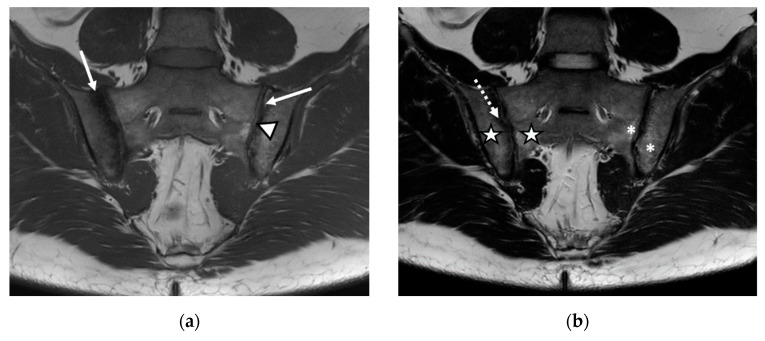

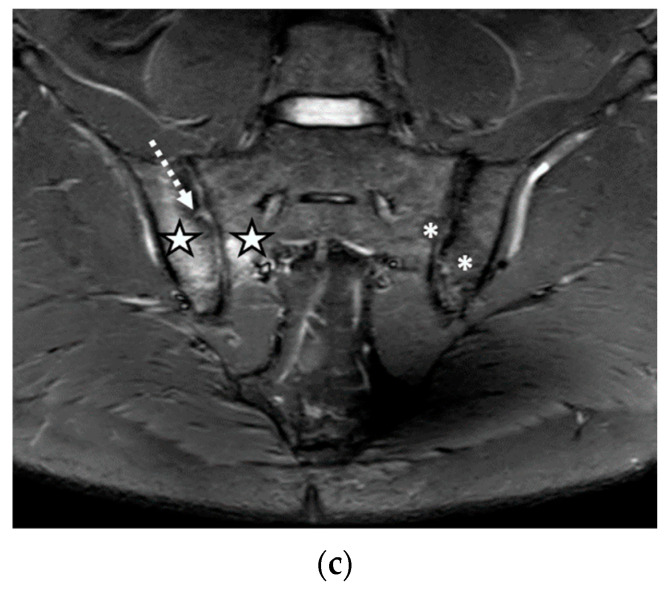
The MRI aspect of the sacroiliac joints included pseudo-widening of both sacroiliac joints, with marked bone marrow edema (star) of both the sacrum and the right iliac bone, in the proximity of the ipsilateral sacroiliac joint space, right iliac bone erosions (discontinuous arrow), bilateral subchondral sclerosis (continuous arrow), and fat metaplasia (asterisk), affecting the left iliac bone, and the left part of the sacrum and backfill (arrowhead) of the left sacroiliac joint space: (**a**) T1-weighted sequence; (**b**) T2-weighted sequence (without fat suppression); (**c**) STIR sequence.

We evaluated 68 sacroiliac joints. The prevalence of MRI-detected abnormalities is presented in Table 2. The results of the SR were considered as the reference data. Inflammatory and structural changes were detected on the MRI sequences of the sacroiliac joints. The most frequently encountered inflammatory change, both on the left and right sacroiliac joint, was the presence of bone marrow edema (44.11–58.82%), followed by enthesitis (17.64–26.47%) and synovitis (11.76–17.64%). Regarding structural changes, the most prominent was subchondral sclerosis (50–59.37%), followed by erosions (35.29–41.17%), joint space narrowing (38.23%), fat metaplasia (17.64–23.25%), and backfill (11.76–20.58%) (Figure 1, Figure 2 and Figure 3).

The MRI findings on the total number of sacroiliac joints, as well as the interobserver agreement results, are depicted in Table 3.

Moderate to substantial agreement (0.489–0.766) was encountered between the observers regarding the inflammatory changes, whereas poor to almost perfect agreement (0.396–0.813) was noted in the case of structural damage. The overall κ was 0.7 for the JR (substantial agreement), 0.707 for the SRh (substantial agreement), and 0.601 for the JRh (moderate agreement), in comparison with the SR. Regarding the overall inflammatory changes, the SRh and JR showed substantial agreement (κ = 0.708 and 0.742, respectively) with the SR, while the JRh showed moderate agreement (κ = 0.607). The structural damage observed by the JR showed substantial agreement (κ = 0.676) with the SR, while the SRh and JRh had substantial and moderate agreement (κ = 0.705 and 0.596, respectively) with the SR.

### Associations between Clinical and MRI Findings

A positive Patrick’s maneuver on the right was associated with the presence of synovitis on the MRI interpreted by the SR (*p* = 0.019), while a positive Patrick’s maneuver on the left was associated with the presence of bone marrow edema interpreted by the SR (*p* = 0.019). None of the other parameters tested, such as enthesitis, subchondral sclerosis, erosions, backfill, fat metaplasia, and joint narrowing, reflected an association with Patrick’s maneuver.

The disease activity score ASDAS-CRP showed an association with fat metaplasia, both on the right (*p* = 0.012) and on the left (*p* = 0.034) sacroiliac joints, and also with joint space narrowing of the right (*p* = 0.007) and left (*p* = 0.007) sacroiliac joints. None of the other MRI parameters tested, such as bone marrow edema, synovitis, enthesitis, subchondral sclerosis, erosions, and backfill, showed associations with the disease activity score.

## 4. Discussion

The use of MRI has significantly improved the assessment and management of patients with SpA. As part of the updated New York criteria for AS, the presence of bilateral moderate or unilateral severe radiographic sacroiliitis was previously required for diagnosis. This typically caused a 7–10-year delay in diagnosis [15]. MRI is of great importance in assessing the diagnosis of AS, but also in monitoring the disease activity through the presence of inflammatory/acute findings, and in monitoring the structural joint damage and progression of the disease [16,17].

In our study, the senior radiologist and senior rheumatologist showed substantial agreement (the overall κ = 0.707) in interpreting the MRI of the sacroiliac joints, in concordance with a study conducted by Rueda et al. Their study included an expert radiologist, a local radiologist, and a rheumatologist as observers, showing substantial agreement between the expert radiologist and the rheumatologist. In our case, the senior radiologist and rheumatologist had special training in interpreting MRIs in SpA [18].

A study by Arnbak et al., regarding the back pain cohort in Denmark, showed moderate to almost perfect interobserver agreement, with the almost perfect agreement being found for bone marrow edema. In our study, the interobserver reliability between the senior radiologist, senior rheumatologist, and junior radiologist was substantial, while moderate agreement was found between the senior radiologist and junior rheumatologist [19].

In a study by Geijer et al., regarding findings of sacroiliitis on computed tomography (CT) scans, the authors showed substantial agreement between the interpretations of two expert radiologists; whereas, in our study, the SR and JR had substantial agreement in reading the scans [20].

A study by Berg et al. evaluated the patients included in the DESIR cohort. The images were interpreted by local rheumatologists or radiologists, versus central trained readers. In the case of recent-onset inflammatory back pain, the trained readers and local rheumatologists/radiologists showed substantial agreement, in concordance with our study, in which the SR, SRh, and JR showed substantial agreement. Compared with this study, our readers were blinded to the diagnosis [21].

Overall, our study demonstrated that the senior radiologist, senior rheumatologist, and junior radiologist trained by the senior were in substantial agreement regarding inflammatory changes (κ = 0.708—SRh and κ = 0.748—JR), structural damage (κ = 0.676—SRh and κ = 0.705—JR), and overall findings (κ = 0.707—SRh and κ = 0.70—JR). The junior rheumatologist demonstrated moderate agreement with the senior radiologist regarding inflammatory, structural, and overall changes (κ = 0.607, κ = 0.596, and κ = 601, respectively). The findings may prove that, with special training, the results between radiologists and rheumatologists are comparable.

Even if there was substantial agreement between the SR, SRh, and JR, regarding inflammatory, structural, and overall sacroiliac joint abnormalities, there were certain differences between the interpretations. One reason for these discrepancies is related to the fact that the interpretation of an MRI is subjective, dependent on the interpreter. Another reason may be that the SR and JR have more experience in interpreting MRIs in general, having opportunities to describe MRI lesions on a daily basis. Due to the fact that the SRh underwent special training in spondyloarthritis imaging, the interobserver agreement was substantial. Regarding the moderate agreement with the JRh, it only proves that the better the training, the better the agreement between interpreters.

In our study, we also encountered a positive association (*p* = 0.019) between bone marrow edema and clinical maneuvers, regarding the pain of the sacroiliac joints (Patrick’s maneuver); these findings were compatible with a study conducted by Yang et al., which proved that bone marrow edema correlated with pain in the sacroiliac joints [22].

In addition, the presence of synovitis was associated with a positive Patrick’s maneuver (*p* = 0.019), with both bone marrow edema and synovitis being inflammatory changes that can trigger positive provocation maneuvers of the sacroiliac joints.

In a study by Castro et al., which aimed to assess the construct validity of clinical tests to identify sacroiliac joint inflammation in patients with nr-axSpA, the authors concluded that the FABER test (Patrick’s) showed greater likelihood ratios to identify sacroiliac joint inflammation, in concordance with our findings. In our case, active/inflammatory changes had a positive correlation with Patrick’s maneuver [23].

In our study, we showed a positive association between fat metaplasia on both sacroiliac joints (*p* = 0.017—right; *p* = 0.017—left) and the sacroiliac joint space narrowing on both sides (*p* = 0.012—right; *p* = 0.034—left). The evidence in the literature is contrasting, with MRI scores showing either no association with ASDAS-CRP or a positive association. We believe that these discrepancies may be due to the fact that inflammation parameters, such as CRP, are not frequently high in spondyloarthritis, even if the patients are proven to have high disease activity from a clinical point of view [24,25,26].

The limitations of our study were the small sample size and the fact that the interpreters were aware of the overall diagnosis of the patients, but not of the clinical and paraclinical data.

## 5. Conclusions

Our study showed substantial agreement between the senior radiologist, senior rheumatologist, and junior radiologist, and moderate agreement with the junior rheumatologist; these findings are in concordance with other studies in the literature. However, the interobserver reliability in the MRI assessment of sacroiliac joints is not a very well-studied domain, so further studies on larger numbers of patients have to be conducted in order to establish the level of agreement.

## Figures and Tables

**Table 1:** Demographic and clinical features of the patients.

Demographic and Clinical Features Patients (n = 34)	Data
Sex (patients, %)	29 males (85.29%); 5 females (14.79%)
Age (years) (mean, SD)	39.14 (10.31)
Disease duration (months) (mean, SD)	14.57 (13.25)
Patrick’s maneuver (patients, %)	
Right	28 patients (82.35%)
Left	25 patients (73.52%)
VAS (mm) (mean, SD)	
Right	72.3 (11.8)
Left	71.1 (10.2)
ESR (mm/h) (mean, SD)	46.55 (23.49)
CRP (mg/L) (mean, SD)	30.29 (23.22)
BASDAI (mean, SD)	7.34 (1.57)
ASDAS-CRP (mean, SD)	4.67 (1.24)
BASFI (mean, SD)	7.24 (1.64)

**Table 2:** Magnetic resonance imaging (MRI) findings of the left and right sacroiliac joints, obtained by the senior radiologist, junior radiologist, senior rheumatologist, and junior rheumatologist—the number and percentage of positive cases for each abnormality.

MRI Findings	Senior Radiologist n(%)	Junior Radiologist n(%)	Senior Rheumatologist n(%)	Junior Rheumatologist n(%)
LEFT (n = 34)				
Bone marrow edema	15 (44.11)	13 (38.24)	14 (41.18)	10 (29.41)
Synovitis	4 (11.76)	3 (8.82)	2 (5.88)	2 (5.88)
Enthesitis	9 (26.47)	5 (14.71)	6 (17.65)	5 (14.71)
Subchondral sclerosis	17 (50)	13 (38.24)	14 (41.18)	17 (50)
Erosions	14 (41.18)	15 (44.12)	13 (38.24)	10 (29.41)
Backfill	7 (20.59)	4 (11.76)	5 (14.71)	3 (8.82)
Fat metaplasia	6 (17.65)	7 (20.59)	6 (17.65)	4 (11.76)
Joint space narrowing	13 (38.24)	10 (29.41)	12 (35.29)	8 (23.53)
RIGHT (n = 34)				
Bone marrow edema	20 (58.82)	14 (41.18)	13 (38.24)	9 (26.47)
Synovitis	7 (20.59)	3 (8.82)	4 (11.76)	2 (5.88)
Enthesitis	6 (17.65)	4 (11.76)	6 (17.65)	5 (14.71)
Subchondral sclerosis	19 (55.88)	17 (50.00)	15 (44.12)	17 (50.00)
Erosions	12 (35.29)	9 (26.47)	13 (38.24)	8 (23.53)
Backfill	4 (11.76)	3 (8.82)	3 (8.82)	2 (5.88)
Fat metaplasia	8 (23.53)	6 (17.65)	5 (14.71)	5 (14.71)
Joint space narrowing	13 (38.24)	11 (32.35)	10 (29.41)	8 (23.53)

**Table 3:** Global MRI findings for the 68 sacroiliac joints and the level of agreement between readers. SR—senior radiologist; JR—junior radiologist; SRh—senior rheumatologist; JRh—junior rheumatologist.

MRI Findings n = 68	SR n(%)	JR n(%)	SRh n(%)	JRh n(%)	SR vs. JR κ Agreement	SR vs. SRh κ Agreement	SR vs. JRh κ Agreement
Bone marrow edema	35 (51.47)	27 (39.71)	27 (39.71)	19 (27.94)	0.766 substantial	0.709 substantial	0.535 moderate
Synovitis	11 (16.18)	6 (8.82)	6 (8.82)	4 (5.88)	0.668 substantial	0.535 moderate	0.489 moderate
Enthesitis	15 (22.06)	9 (10.29)	12 (17.65)	10 (14.71)	0.64 substantial	0.716 substantial	0.7 substantial
Subchondral sclerosis	36 (52.94)	30 (44.12)	29 (42.65)	34 (50)	0.649 substantial	0.679 substantial	0.647 substantial
Erosions	26 (38.24)	24 (35.29)	26 (38.24)	18 (26.47)	0.747 substantial	0.813 almost perfect	0.603 moderate
Backfill	11 (16.18)	7 (10.29)	8 (11.76)	5 (7.35)	0.492 moderate	0.649 substantial	0.402 poor
Fat metaplasia	14 (20.59)	13 (19.12)	11 (16.18)	9 (13.24)	0.55 moderate	0.43 moderate	0.396 poor
Joint space narrowing	26 (38.42)	21 (30.88)	22 (32.35)	16 (23.53)	0.709 substantial	0.743 substantial	0.597 moderate
